# HIV-Response Intergenerational Participation Intervention Among Black Men in Ontario, Canada: Protocol for a Pilot Intervention Study

**DOI:** 10.2196/48829

**Published:** 2023-07-11

**Authors:** Egbe B Etowa, Priscilla N Boakye, Roger Antabe, Josephine Pui-Hing Wong

**Affiliations:** 1 Ontario HIV Treatment Network Toronto, ON Canada; 2 Daphne Cockwell School of Nursing Toronto Metropolitan University Toronto, ON Canada; 3 Department of Health and Society University of Toronto Scarborough, ON Canada

**Keywords:** community-based participatory project, heterosexual Black men, HIV response, intergenerational participation, intervention

## Abstract

**Background:**

Black men and their communities are more affected by HIV. Although they constitute less than 5% of the Ontarian population, they accounted for 26% of new HIV diagnoses in 2015, nearly half of which (48.6%) were attributed to heterosexual contact. HIV stigma and discrimination reinforce African, Caribbean, and Black men’s HIV vulnerability by creating unsafe environments that deter them from testing and disclosure, resulting in isolation, depression, delayed diagnosis and linkage to treatment and care, and poor health outcomes. In response to these challenges, intergenerational strategies were identified from previous community-based participatory studies as best practices to reduce HIV vulnerabilities and promote resilience among heterosexual Black men and communities. The proposed intervention is premised on this recommendation of intergenerational intervention.

**Objective:**

The overarching objective is to engage heterosexual Black men and communities in cocreating a community centered, culturally safe intergenerational intervention to reduce HIV vulnerabilities and related health disparities.

**Methods:**

We will engage 12 diverse community stakeholders in Ontario, inclusive of heterosexual Black men, in 8 weekly sessions to evaluate existing evidence of effective HIV health literacy interventions, identify essential and relevant aspects, and work collaboratively to co-design the HIV-Response Intergenerational Participation (HIP) intervention for use with Black men and communities. Next, we will recruit 24 self-identified heterosexual Black men aged 18-29, 29-49, and ≥50 years. We will pilot and evaluate the HIP intervention with 24 heterosexual Black men from these 3 age groups (split as 2 events: a total of 12 participants in person in Toronto and 12 participants on the web in Windsor, London, and Ottawa). We will use the data obtained along with questionnaires from validated scales and focus groups to evaluate the effectiveness of HIP. The data will include HIV knowledge, perceived stigma toward people living with HIV, acceptance and uptake of HIV testing, preexposure prophylaxis (PrEP), postexposure prophylaxis (PEP), and condom use. We will also collect data related to perceptions about system-level factors such as discrimination, socially misconstrued masculine identity, etc. Quantitative analysis will essentially be a univariate descriptive analysis. We will use thematic analysis to highlight the results of the focus group discussions. Finally, we will disseminate the evaluation results and engage researchers, leaders, Black men, and communities to expand the project team and scale up the intervention in Ontario and across Canada.

**Results:**

Implementation commences by May 2023, and by September 2023, we should have produced, among others, an evidence-informed HIP intervention that can be adapted for use by heterosexual Black men and communities beyond Ontario.

**Conclusions:**

The pilot intervention will strengthen critical health literacy and build resilience against HIV through intergenerational dialogue among heterosexual Black men of all ages.

**International Registered Report Identifier (IRRID):**

PRR1-10.2196/48829

## Introduction

### Background

Black men and communities are disproportionately affected by HIV through heterosexual exposure than other racialized groups in Canada [1-3]. While Black people make up only 5% of the Ontarian population, they accounted for 26% of new HIV diagnoses in 2015, with nearly half (48.6%) attributed to heterosexual contact [4-6]. Black men remain vulnerable to HIV due to structural inequities, including the lack of access to culturally safe programs that provide them opportunities to dialogue about their vulnerabilities and cocreate solutions [7-9]. Systemic factors such as institutional discrimination, poor representation of Black populations in health care decision-making, a lack of accessible information in relevant languages, and a lack of culturally safe health services constitute barriers to effective HIV responses [10-13]. Furthermore, HIV stigma and discrimination hinder them from testing and disclosure, thereby increasing their vulnerabilities, social isolation, depression, delays in HIV testing, diagnosis, linkage to treatment or care, and poor health outcomes [14,15]. HIV stigma also reinforces community denial, undermines HIV prevention efforts, impedes the provision of accessible community care, and prevents access to HIV information [16,17].

In 2020, the weSpeak team undertook a community consultation on best practices to reduce HIV vulnerabilities among heterosexual Black men and communities. They engaged 60 heterosexual Black men in Ontario through *group concept mapping* [18,19]. Data analysis of the 123 statements generated by the participants showed that the actionable cluster *“individual and family level HIV prevention”* was ranked highest among all the go-zone statements in both the scores of importance (4.06/5.0) and feasibility (3.61/5.0). Furthermore, detailed reviews of the statements within this cluster indicated the need for older generations to meaningfully engage the younger generations within families and in communities to promote cultural pride, collective resilience, and positive action to reduce HIV vulnerabilities. The priority of developing intergenerational programs and safe spaces for heterosexual Black men to engage in culturally safe dialogue and action was also supported by findings from the qualitative phase of weSpeak where over 200 heterosexual Black men and service providers were engaged in focus groups and individuals interviews in 2019 [20-24]. Emerging from these interviews was the call by participants to use an intergenerational approach to engage heterosexual Black men, families, and community stakeholders to raise awareness about HIV prevention and increase connections to HIV care as a community priority.

Evidence from intervention research undertaken in the United States also shows that parent-, family-, and community-engaged HIV prevention programs are effective (eg, the *Collaborative HIV Prevention and Adolescent Mental Health Project*) [25-27]. Intergenerational interventions such as youth-adult partnerships and parent-youth connections have been shown to build connection, improve youth efficacy, increase capacities for engagement, and increase responsibility at individual and systemic levels. They spur social-level benefits such as adult support, encouragement, shared trust, shared experience, and knowledge, and these in turn, further produce individual level outcomes of self-efficacy, self-esteem, and self-confidence. The cycle continues with individual outcomes leading to more social outcomes, such as positive peers, and a system-level outcome, for example, education of others [28-31]. Hence, models that support parent-youth and intergenerational dialogue and connections are promising for HIV-related intervention [32].

### Objectives

In this proposed study, we will draw on existing literature and local evidence [17,20-24] to engage heterosexual Black men and communities to co-design the HIV-Response Intergenerational Participation (HIP) intervention*.* Based on the recommendations from weSpeak concept mapping, intergenerational connection is conceptualized beyond individual families to include social connections across different generations of heterosexual Black men and leaders in Black communities. The overarching goal of the study is to promote literacy on HIV prevention (eg, condom use, preexposure prophylaxis [PrEP], postexposure prophylaxis [PEP], safer sex negotiation, partner communication), testing (eg, access, point-of-care testing, and self-testing), and linkage to care. The specific objectives are to:

1. Advance critical HIV health literacy among heterosexual Black men and communities.
2. Engage heterosexual Black men, community leaders, and stakeholders in co-designing the HIP intervention to reduce HIV vulnerabilities and promote community resilience.
3. Pilot the HIP intervention with heterosexual Black men of diverse ages and assess its acceptability, feasibility, and effectiveness.
4. Apply study results to refine the HIP intervention for potential scale-up in Ontario and Canada.

### Theoretical Framework

The project is underpinned by the principles of health equity, community empowerment, and meaningful engagement [33,34]. We will use a community-based participatory research (CBPR) approach to promote *critical health literacy* among heterosexual Black men and communities [35]. Critical health literacy refers to “the degree to which people are able to access, understand, appraise, and communicate information to engage with the demands of different health contexts in order to promote and maintain good health across the course of life” [36]. Early conceptualization of health literacy mainly focused on *functional health literacy skills*, that is, individuals’ ability to obtain relevant health information to assess their health risks and engage in relevant health behaviors. Over the years, health literacy has evolved to include *interactive health literacy skills*, that is, individuals’ ability to extract information from different sources to make health decisions and interact with service providers with confidence; and *critical health literacy skills,* that is, individuals’ ability to analyze health information to gain a critical understanding of health in the context of social, economic, and environmental determinants and engage in organized efforts to advocate for health equity [37,38]. Critical health literacy has also led to more attention being placed on the roles and responsibilities of health providers and health organizations in providing accessible, culturally safe, and inclusive health information and resources. In using an intergenerational approach to build critical health literacy, heterosexual Black men will be able to tap into community wisdom from key stakeholders (eg, Black men living with HIV, Black knowledge holders, and Black activists for health). A co-designed intervention based on lived experiences and community wisdom will promote collective self-determination among heterosexual Black men and increase community literacy on HIV prevention and care. Guided by *a CBPR approach*, we will apply meaningful engagement strategies throughout the study. We will set up a project advisory committee (PAC) comprising 6-8 Black leaders from the youth, heterosexual Black men living with HIV, LGBTQ+ (lesbian, gay, bisexual, transgender, and queer/questioning), faith, and social justice sectors. The PAC will advise the team on cultural relevance and inclusion, research design and processes, community connections, data interpretation, and knowledge dissemination. Using CBPR, we will build equitable collaborative partnerships that promote meaningful involvement of people, organizations, and communities to increase collective self-determination in Black health, enhance HIV prevention, and improve health outcomes in heterosexual Black men and communities [14-17]. The processes will empower heterosexual Black men and the community to gain a critical understanding of the interpersonal and structural determinants of HIV vulnerabilities and codevelop strategies to address HIV-related health disparities experienced by heterosexual Black men and communities.

## Methods

The proposed project consists of 4 interconnected components that work in tandem to engage heterosexual Black men and communities in knowledge uptake and cocreate a culturally safe intergenerational HIV literacy intervention.

### Component One: Engaging Heterosexual Black Men in Knowledge Interpretation and Uptake (Objectives 1 and 2)

#### Overview

The objective of Component One is to apply an integrative approach to engage heterosexual Black men in knowledge interpretation and uptake to inform the development of the HIP intervention. We will recruit 12 self-identified heterosexual Black men with diverse lived experiences. Participation criteria include being aged 18 years or older; being HIV-positive, HIV-negative, or having an unknown HIV status; engagement in faith, arts, media, health or social care, education, or social justice sectors; being interested in codeveloping culturally safe and accessible HIV health literacy intervention for heterosexual Black men and communities; and being available to take part in 2 series of 4 co-design workgroup sessions. To achieve our objective of developing an intergenerational HIP intervention, we will specifically recruit heterosexual Black men from 3 age groups: 18 to 29 years; 30 to 49 years; and 50 years or older. We will engage participants in 4 weekly sessions on critical interpretation of evidence [20-24] to create a shared understanding of HIV vulnerabilities of heterosexual Black men at multiple levels: intrapersonal, interpersonal, organizational, and policy levels. The shared critical understanding gained through the following 4 sessions will be used to inform the co-design of the HIP intervention in Component Two.

#### Session 1: Social Identities of Heterosexual Black Men and HIV Vulnerabilities

In this session, we will present existing evidence on how heterosexual Black men negotiate their racialized masculine identities and social positions (race or ethnicity, age groups, socioeconomic status, education, employment, relationship status, etc) through practices that may protect them from HIV and put them at increased risk of HIV. We will then engage participants to identify real-world and culturally relevant strategies to address these issues.

#### Session 2: Structural Determinants of HIV Vulnerabilities of Heterosexual Black Men

In this session, we will present existing evidence on how structural determinants and public policies impact the HIV vulnerabilities of heterosexual Black men through inaccessibility to HIV services and other health and social care resources, socioeconomic resources, and adequate health care access (access to employment, stigma, and discrimination, etc). We will then engage participants to identify real-world and culturally relevant strategies to address these issues.

#### Session 3: Gaps in HIV Response and Cascade of HIV Care Affecting Heterosexual Black Men and Communities

In this session, we will present local evidence on existing heterosexual Black men’s experiences in accessing HIV prevention, testing, and care in Ontario. In addition, we will invite 3 heterosexual Black men panel speakers with lived experiences to share their insights about gaps in HIV response. We will then engage participants to identify strategies to address these issues.

#### Session 4: Critical HIV-Related Health Literacy for Change

In this session, we will engage participants in learning and critically examining the 3 levels of critical health literacy: (1) functional health literacy skills, that is, heterosexual Black men’s ability to obtain relevant and accurate information on HIV prevention (eg, transmission, condom use, PrEP, and PEP; treatment as prevention; undetectable=untransmissible, etc) and connection to care (eg, HIV testing, early diagnosis, and treatment and care) to guide their actions and behaviors; (2) interactive health literacy skills, that is, heterosexual Black men’s ability to extract information from different sources (eg, evidence on social determinants of HIV vulnerabilities, Black pride as a protective factor, social support and resilience, etc) to make HIV related sexual and health decisions, and to interact with service providers with confidence to meet their health needs; and (3) critical health literacy*,* that is, heterosexual Black men’s ability to analyze population health information (eg, proportion of HIV infections among heterosexual Black men compared to other groups; impact of gender discourses on sexual practices) to gain a critical understanding of HIV vulnerabilities in the context of social, economic, and environmental determinants, and to engage in organized efforts to advocate for health equity. We will hire an intervention specialist to work closely with the study team and participants in Component One to ensure that critical information and key elements for co-design are documented and applied in Component Two. Results from Component One will be presented to the PAC for feedback and discussion to inform Component Two.

### Component Two: Co-design of HIP Intervention (Objectives 1 and 2)

The aim of this component is to apply the results from Component One to develop the HIP intervention and to seek community feedback on the intervention before pilot testing it in Component Three. We will engage the same 12 participants from Component One in the co-design of the HIP intervention. Participants who are not able to continue due to time constraints or other reasons will be replaced by new participants with detailed preparation based on the results of Component One. There will be *4 sessions* of the HIP intervention co-design:

1. In *session 1,* participants will collaborate to define the intervention objectives and outcome indicators.
2. In *session 2*, participants will identify the contents, learning activities, and required resources.
3. In *session 3,* participants will identify evaluation measurements (tools and indicators). After session 3, the team will work closely with the intervention specialist to construct the HIP intervention based on results and recommendations from participants. This semifinal HIP intervention will be presented to the PAC for feedback.
4. In *session 4*, participants will critically review the HIP intervention and collaborate to finalize HIP. The expected outputs of Component Two include 4-6 intervention modules with a facilitation manual, participant worksheets, experiential learning resources, and community engagement strategies. The HIP intervention modules will be designed based on the 3 levels of critical health literacy, as described above. In addition, we will produce a training toolkit for HIP champions with clear training objectives, theoretical concepts, demonstration videos, and implementation fidelity checklists to ensure that the intervention is implemented consistently.

### Component Three: Piloting the HIP Intervention and Evaluation (Objective 3)

#### Piloting

In Component Three, we will pilot the HIP intervention co-designed by participants in Components Two and Three. We will engage 2 groups of 12 heterosexual Black men (N=24): a total of 12 participants for in-person piloting in Toronto and another 12 participants for virtual piloting from all sites (a total of 3 per site). Based on our team’s previous experience in intervention research, we anticipate that the HIP intervention will consist of 6 half-day sessions or eight 2-hour sessions (a total of 16-18 hours). Participation criteria include being self-identified as heterosexual Black men; being aged 18 years or older; being interested in HIV literacy and becoming an HIV prevention champion among heterosexual Black men and communities; and being available to take part in 16-18 hours of colearning in group sessions. To achieve our objective of implementing an intergenerational HIP intervention, we will specifically recruit heterosexual Black men from 3 age groups: 18 to 29 years, 30 to 49 years, and 50 years or older to engage in the HIP intervention. The HIP sessions will be cofacilitated by EBE and JPW in person and RA and PNB on the web. Piloting HIP as both an in-person and a web-based intervention will enable us to gain important insights for future scale-up. For example, the web-based format will enable us to engage heterosexual Black men across regions and increase access to participation among heterosexual Black men living in smaller communities.

#### Evaluation

All participants will be asked to complete a presurvey with sociodemographic information and questions on their knowledge of HIV transmission and prevention, community resources on HIV responses for heterosexual Black men and communities, structural determinants of HIV, stigma attitude, HIV testing behaviors, levels of community engagement, and readiness for HIV championship in Black communities. We will also invite participants to complete a survey at immediate-, post-, and 8-week post-HIP intervention to evaluate the effectiveness of HIP at all 3 levels of critical HIV health literacy. Depending on the final design of HIP, some of the relevant scales may include the HIV Knowledge Questionnaire [39], the adapted Social Capital Scale [40], the Multidimensional Inventory of Black Identity (MIBI) [41], and the Scale of Stigmatizing Attitudes Toward People Living with HIV (SAT-PLWHA-S) [42,43]. In addition, we will collect postsession feedback forms at each training session to assess participants’ self-report of satisfaction, knowledge gained, and confidence in specific skills. Insights gained from the postsession feedback will be used to refine and improve HIP for future scale-up.

#### Data Analysis

Because the survey is for a small-sized sample (N=24), quantitative analysis will essentially be a univariate descriptive analysis with frequencies, percentages, and central tendencies (mean, median, mode, and range). We will use Braun and Clarke’s [44] 6-step framework to conduct thematic analysis of the qualitative data from focus groups.

### Component Four: End-of-Grant Knowledge Translation and Evidence Uptake (Objectives 1 and 4)

**Figure 1:**
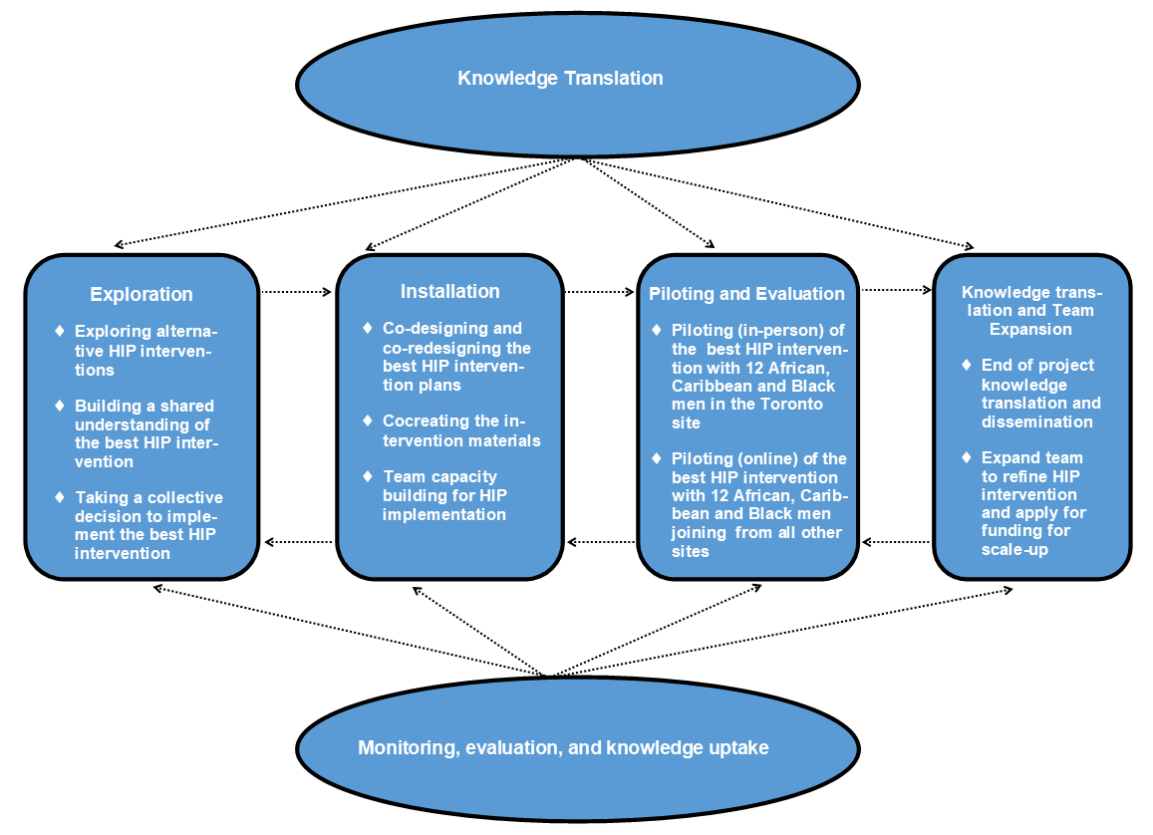
Adapted Fixen’s interconnected phases of implementation in HIP Project. HIP: HIV-response intergenerational participation.

In addition to the knowledge translation activities integrated throughout phases 1-3, we will be engaged in end-of-grant knowledge translation in 2 ways. First, we will expand our existing research team to engage additional community partners, interdisciplinary researchers, and knowledge users to form a new team to refine and scale up the HIP intervention for use across Ontario and Canada. Second, we will engage community partners and collaborators to coproduce and disseminate culturally inclusive, accessible, and audience-specific knowledge translation products (eg, infographics, e-posters, community reports, conference presentations, peer-reviewed publications, community forums to demonstrate HIP intervention activities, webinars, and social media postings). Figure 1 summarizes the interconnected relationships of all 4 study components [45].

### Informed Consent

Participants who meet the inclusion criteria and express interest in participating will receive a consent form with detailed information describing the purpose of the study, the data collection process, anticipated risks and benefits, and the process of maintaining confidentiality. The consent will have principal investigator’s contact information for participants to reach out to if they have questions or are seeking additional information about the study. Participants will be given a week to review the consent form to enable them to make an informed decision whether they want to participate. As an ongoing process, we will continually seek consent from participants during the implementation of each component of the project.

### Team Expertise and Resources

Our interdisciplinary team consists of HIV researchers, service providers, community organizations, faith organizations, and heterosexual Black men. The team consists of researchers of diverse career stages (full professors, associate professors, assistant professors, postdoctoral fellows, research coordinators, and research assistants). The research team brings extensive relevant experience in HIV community-based research, knowledge mobilization, translating research evidence into community-based interventions, intergenerational intervention, heterosexual Black men studies, equity-based health research, etc. The team also includes community collaborators and partners, with capacities to contribute to project advisory, knowledge translation, and the design, delivery, and scaling up of the pilot intervention.

### Potential Limitations and Strengths

We recognize 2 potential challenges in carrying out this research. First, as a catalyst project, we are testing out novel ideas over a 12-month period. To ensure that we achieve our objectives, we will establish an implementation plan with clear time lines and deliverables. We will also work closely with our knowledge users and collaborators to ensure successful community engagement. Second, our research activities require participants to engage in a series of 4-6 group sessions. We anticipate that some participants may withdraw due to other competing demands. We will overrecruit, that is, a total of 12 participants per group instead of 8-10 to ensure sustainable participation. In addition, we will design our group activities in ways that promote enthusiasm and commitment, which our team has achieved in previous studies.

### Ethics Approval

This study was reviewed and approved by the University of Toronto HIV Research Ethics Review Board (RIS Protocol #44285), the Toronto Metropolitan University Research Ethics Board (REB 2023-099), and the University of Ottawa Research Ethics Board.

## Results

This is a funded 1-year project with a funding start date of September 2022. Due to the time lag for research ethics approval, data collection is proposed to commence in July 2023, with an expected project end date of March 2024. To date, recruitment has been limited to 12 members of the PAC who are providing insights into the project planning activities. Actual recruitment of participants will commence in July 2023 prior to data collection. We expect that our innovative, action-oriented project will produce numerous deliverables, including (1) documentation of effective processes to engage heterosexual Black men and community stakeholders in co-designing the HIP intervention; (2) knowledge and insights gained from piloting and evaluating the HIP intervention; (3) an evidence-informed HIP intervention that can be adapted for use by heterosexual Black men and communities beyond Ontario; (4) accessible knowledge translation products for community members as well as researchers and policy makers; and (5) an expanded team to refine HIP and secure resources to scale-up HIP across Ontario and Canada. We also anticipate many positive outcomes. At the individual level, the expected outcomes include increased capacity among heterosexual Black men to acquire accurate knowledge on HIV prevention and increased understanding of the structural influences on Black masculinities and sexualities; apply knowledge to guide their sexual practices and help-seeking behaviors; and gain increased skills in communicating their needs for HIV resources to service providers. At the collective level, we anticipate increased engagement and collaboration among heterosexual Black men, families, and communities to promote open dialogue about sexualities, HIV prevention and care, cultural pride, and collective resilience. There will also be increased capacity among heterosexual Black men and communities to advocate for a culturally inclusive HIV response and equitable access to related care and resources [46,47].

## Discussion

The HIP is an innovative project that recognizes that heterosexual Black men’s vulnerability and resilience are not an individualized phenomenon but a function of the social system in which they live. Based on this fact, HIP proposes to apply the collaborative efforts of heterosexual Black men themselves and members of their social system (leaders, service providers, intervention specialists, decision makers, and policy makers, community-based research experts, women, local businesses, the LGBTQ community, people living with HIV, etc) to co-design a culturally safe intervention. The pilot intervention will strengthen critical health literacy and build resilience against HIV vulnerabilities through intergenerational dialogue among heterosexual Black men of all ages without reinforcing any preexisting stereotype and discrimination against any other group in their social system.

## Appendix

Multimedia Appendix 1 Peer-review report by HIV/AIDS Community-Based Research - General Stream (Merged) (CIHR/IRSC, Canada).

## Abbreviations

CBPR: community-based participatory research
HIP: HIV-response intergenerational participation
LGBTQ+: lesbian, gay, bisexual, transgender, and queer/questioning
MIBI: Multidimensional Inventory of Black Identity
PAC: project advisory committee
PEP: post-exposure prophylaxis
PrEP: pre-exposure prophylaxis
SAT-PLWHA-S: Scale of Stigmatizing Attitudes Toward People Living with HIV

## Data Availability

The anonymized and encrypted data generated by the study will be stored on a secure institutional repository for a period of 5 years for the purpose of conducting secondary analysis by researchers upon reasonable request.
