# Supplementary material for: HIV-Response Intergenerational Participation Intervention Among Black Men in Ontario, Canada: Protocol for a Pilot Intervention Study
Source: JMIR Res Protoc. 2023 Jul 11;12:e48829. doi: 10.2196/48829 (PMC10369308; doi:10.2196/48829)
Supplement: Multimedia Appendix 1 [file resprot_v12i1e48829_app1.pdf]

Review Type/Type d'évaluation: SO Notes /Notes de l'agent scientifique
Name of Applicant/Nom du chercheur: Etowa, Egbe Bassey
Application No./Numéro de demande: 483731
Agency/Agence: CIHR/IRSC
Competition/Concours: 2022-04-26 Catalyst Grant: HIV/AIDS and STBBI Community-Based Research/Subvention catalyseur : Recherche communautaire sur le VIH/SIDA et ITTS
Committee/Comité: HIV/AIDS Community-Based Research - General Stream (Merged)/Recherche communautaire sur le VIH/SIDA - volet général
Title/Titre: HIV-Response Intergenerational Participation (HIP) Intervention among Heterosexual Black Men and Communities in Ontario, Canada

Assessment/Évaluation:

Strengths:

The reviewers were encouraged that this is a very unique project which recognizes the prevalence of HIV in ACB communities in Ontario. The focus on Black men being involved in designing and interpreting the research added to the strength of the research, as well as demarcating three age groups and intergenarationality as important demographic factors for HIV vulnerability.

The qualifications of the project team as well as their strong community support are valuable assets for this research proposal. Reviewers were also happy that the project arose from ongoing community work and that the research built in a layer for mentorship for new comers.

Weaknesses:

The reviewers needed to know more about the levels of HIV literacy among Black men in addition to a discussion on barriers to HIV health promotion. There needs to be some information tying change in HIV literacy measure to behaviour change. Reviewers also felt that the proposed study did not take into account the change in intergenerational dynamics post immigration. It was believed that strategies that have proved successful in the home country may not be pertinent post migration (that is, in Canada).

It appears that the inclusion criteria for mentoring was based on age only however reviewers surmised that other aspect of participant’s life should be considered. Unique geographical differences, sociodemodemographic variables considered could result in a counterproductive study approach. The Black community is heterogeneous and these aspects of diversity should be considered.

The study does not show an intent for capacity building. A gap analysis for capacity building is needed, particularly with the intergenerational approach. It shouldn’t be taken for granted that the older generation has the skills to provide mentorship. This may be particularly challenging in larger communities such as Toronto.

The study has a very busy research schedule which may be taxing on the participants. Clarity is also needed on whether the 6 individuals who are being recruited for the advisory role are different from the 12 study participants. Further, reviewers needed to know whether the advisory committee will participate in

**Review Type/Type d'évaluation:**	SO Notes /Notes de l'agent scientifique
**Name of Applicant/Nom du chercheur:**	Etowa, Egbe Bassey
**Application No./Numéro de demande:**	483731
**Agency/Agence:**	CIHR/IRSC
**Competition/Concours:**	2022-04-26 Catalyst Grant: HIV/AIDS and STBBI Community-Based Research/Subvention catalyseur : Recherche communautaire sur le VIH/SIDA et ITTS
**Committee/Comité:**	HIV/AIDS Community-Based Research - General Stream (Merged)/Recherche communautaire sur le VIH/SIDA - volet général
**Title/Titre:**	HIV-Response Intergenerational Participation (HIP) Intervention among Heterosexual Black Men and Communities in Ontario, Canada

Assessment/Évaluation:

curriculum setting.

There needs to be some information on the 'pre' work (preparation stage) that will be developed prior to the intervention. This may include community engagement, particularly as collaborators were not identified for London. .
Further, some information is needed on how individuals are being prepared for 'critical literacy'.

Budget:

Administrative support budget is heavy
Honorarium is low

****

Note: The final rating of the application, provided in the Notice of Decision (NOD), is the averaged rating of the peer review committee members following the discussion of the application during the committee meeting, and therefore may differ from the ratings provided by the assigned reviewers in their respective reviews.

Remarque : La cote définitive de la demande, qui apparaît dans l'avis de décision, représente la moyenne des cotes accordées par les membres du comité d' évaluation par les pairs après avoir débattu de la demande à la réunion du comité. Elle peut donc différer de celle donnée par les évaluateurs dans leur évaluation respective.

````````````````````````````````````````````````````````````````````````````````````````````````````````````````````````````````

***SO Notes end here.***
````````````````````````````````````````````````````````````````````````````````````````````````````````````````````````````````
